# Methicillin Resistant *Staphylococcus aureus* ST398 in Veal Calf Farming: Human MRSA Carriage Related with Animal Antimicrobial Usage and Farm Hygiene

**DOI:** 10.1371/journal.pone.0010990

**Published:** 2010-06-08

**Authors:** Haitske Graveland, Jaap A. Wagenaar, Hans Heesterbeek, Dik Mevius, Engeline van Duijkeren, Dick Heederik

**Affiliations:** 1 Division of Environmental Epidemiology, Institute for Risk Assessment Sciences, Utrecht University, Utrecht, The Netherlands; 2 Department of Infectious Diseases and Immunology, Faculty of Veterinary Medicine, Utrecht University, Utrecht, The Netherlands; 3 Department of Farm Animal Health, Faculty of Veterinary Medicine, Utrecht University, Utrecht, The Netherlands; 4 Central Veterinary Institute of Wageningen UR, Lelystad, The Netherlands; 5 Julius Center for Health Sciences and Primary Care, University Medical Center, Utrecht, The Netherlands; National Institutes of Health, United States of America

## Abstract

**Introduction:**

Recently a specific MRSA sequence type, ST398, emerged in food production animals and farmers. Risk factors for carrying MRSA ST398 in both animals and humans have not been fully evaluated. In this cross-sectional study, we investigated factors associated with MRSA colonization in veal calves and humans working and living on these farms.

**Methods:**

A sample of 102 veal calf farms were randomly selected and visited from March 2007–February 2008. Participating farmers were asked to fill in a questionnaire (n = 390) to identify potential risk factors. A nasal swab was taken from each participant. Furthermore, nasal swabs were taken from calves (n = 2151). Swabs were analysed for MRSA by selective enrichment and suspected colonies were confirmed as MRSA by using slide coagulase test and PCR for presence of the *mecA*-gene. *Spa* types were identified and a random selection of each *spa* type was tested with ST398 specific PCR. The Sequence Type of non ST398 strains was determined. Data were analyzed using logistic regression analysis.

**Results:**

Human MRSA carriage was strongly associated with intensity of animal contact and with the number of MRSA positive animals on the farm. Calves were more often carrier when treated with antibiotics, while farm hygiene was associated with a lower prevalence of MRSA.

**Conclusion:**

This is the first study showing direct associations between animal and human carriage of ST398. The direct associations between animal and human MRSA carriage and the association between MRSA and antimicrobial use in calves implicate prudent use of antibiotics in farm animals.

## Introduction

The continuing emergence of pathogenic organisms resistant to antimicrobials is a global concern. Infections with antibiotic resistant bacteria have been associated with frequent treatment failure and increased severity of disease [1],[2],[3]. Both human and non-human antimicrobial usage may result in increased occurrence of bacterial resistance [4]. Transfer of antimicrobial resistance (genes) from animals to humans may occur through transmission of zoonotic pathogens or commensals through food or direct contact [3]. Methicillin Resistant *Staphylococcus aureus* (MRSA) has traditionally been considered a nosocomial pathogen. More recently MRSA emerged in the community [5] and since 2003 a specific sequence type of MRSA emerged, which was observed in food producing animals and farmers and is referred to as livestock associated MRSA (LA-MRSA) [6]–[11]. This MRSA sequence type is characterized by being non-typable by use of Pulsed Field Gel Electrophoresis with *Sma*I, and was therefore previously called non-typable MRSA (NT-MRSA). All NT-MRSA belong to one clonal complex, in particular multilocus sequence type 398 (ST398) [5]. A high prevalence of ST398 was found among farm animals, predominantly pigs and veal calves, and people occupationally in contact with these animals [5],[6]. Carriers of MRSA may develop an MRSA (wound) infection after disruption of the skin (scarification) or after surgery and are therefore at risk. Additionally, the impact of this MRSA sequence type on farming communities has been considerable because carriers have been treated in isolation in the health care system as part of the infection control measures: the search and destroy policy, common in low prevalence countries [12]. Many case studies show parallel occurrence of MRSA ST398 in both animals and humans [8], [10], [13]–[15]. Risk factors for carrying MRSA ST398 in both animals and humans have not been fully evaluated. Evidence for animal to human transmission of MRSA is often indirect and based on parallel observations in genetic [16],[17] or resistance patterns [18] among isolates or else indications obtained by *in vitro* experiments [19].

This study focussed on the presence of MRSA among veal farmers, their family members and their animals. Specific attention has been given to associations between the presence of MRSA among animals and humans as well as identification of potential determinants of MRSA occurrence such as antibiotic use, hygiene practices at the farm and farm characteristics.

## Materials and Methods

### Study design

We conducted a cross-sectional study on 102 randomly selected veal calf farms. All farms have been visited from October 2007 to March 2008. Nasal swabs (from both anterior nares) were taken from veal calf growers, family members and employees. All participants were asked to fill in a questionnaire containing items about activities on the farm, intensity and duration of animal contact and MRSA history. Questions on potential confounders such as age, sex and smoking were included. In addition questions on farm structure, antibiotic intake (group- and individual treatments, timing and duration of treatment and kind of antibiotics administered) of the calves and farm hygiene were incorporated. The study protocol was approved by the Medical Ethical Committee of Utrecht University. All participants completed an informed consent.

On each farm the square root of the number of veal calves (a minimum of 10 and a maximum of 25 calves for each farm) was randomly selected and sampled using a sterile cotton-wool swab (Cultiplast®). From each calf, one nasal swab was taken from both nares by rubbing the swab in each nostril. Collecting animal samples was in accordance with the Dutch Law on Animal Health and Welfare. The swabs were immediately transported to the laboratory and processed within four hours after collection.

### Laboratory analysis

The nasal swabs were analysed individually using a pre-enrichment containing Mueller Hinton broth with 6.5% NaCl [20]. After overnight incubation at 37°C, 1 mL of the pre-enrichment was transferred into 9 mL selective enrichment of phenyl red mannitol broth (bioMérieux, France) with 75 mg/L aztreonam and 5 mg/L ceftizoxime followed. Ten µL of the selective enrichment broth was inoculated onto sheep blood agar (Biotrading, The Netherlands) and a MRSA Brilliance™ agar (Oxoid, The Netherlands). All suspected colonies were identified as *S. aureus* using standard techniques: colony morphology and slide coagulase assay with rabbit serum [20]. In addition, the presence of the *mecA* gene was confirmed by PCR as described previously [21]. A random selection of the *mecA*-positive colonies (n = 208) was confirmed to be MRSA by PCR of the *S. aureus* specific DNA-fragment Martineau [22].

For all MRSA human isolates (n = 62), and a random selection of MRSA strains isolated from veal calves with a maximum of three per farm (n = 207), *spa* types were determined. The strains were *spa* typed by sequencing of the repetitive region of the protein A gene *spa* [23]. Data were analyzed by using the Ridom Staphtype software version 1.4 (www.ridom.de/staphtype). A random selection of three strains per *spa* type was tested with ST398 specific PCR [24]. Non-ST398 strains were analysed by multilocus sequence typing (MLST) [25] (www.saureus.mlst.net).

### Data analysis

Statistical analysis was performed using SAS software 9.1. Descriptive analyses were undertaken followed by logistic regression analysis (GLIMMIX procedure) to identify determinants of MRSA carriage on individual level (human/calf). Risk factors for MRSA carriage were first identified by univariate logistic regression analysis. Thereafter, multivariate analysis was done by stepwise, forward entry including factors associated with MRSA positivity in humans and calves (P<0.2). Hierarchical structure of the data was taken into account to adjust for the fact that observations of human and calves on the same farm may not be independent. Relationships between MRSA carriage in humans and veal calves were further studied by assessing the shape of these relationships by means of nonparametric regression modelling (smoothing) using generalized additive models (PROC GAM). A P-value<0.05 was considered statistically significant.

## Results

### Humans

**Figure 1:**
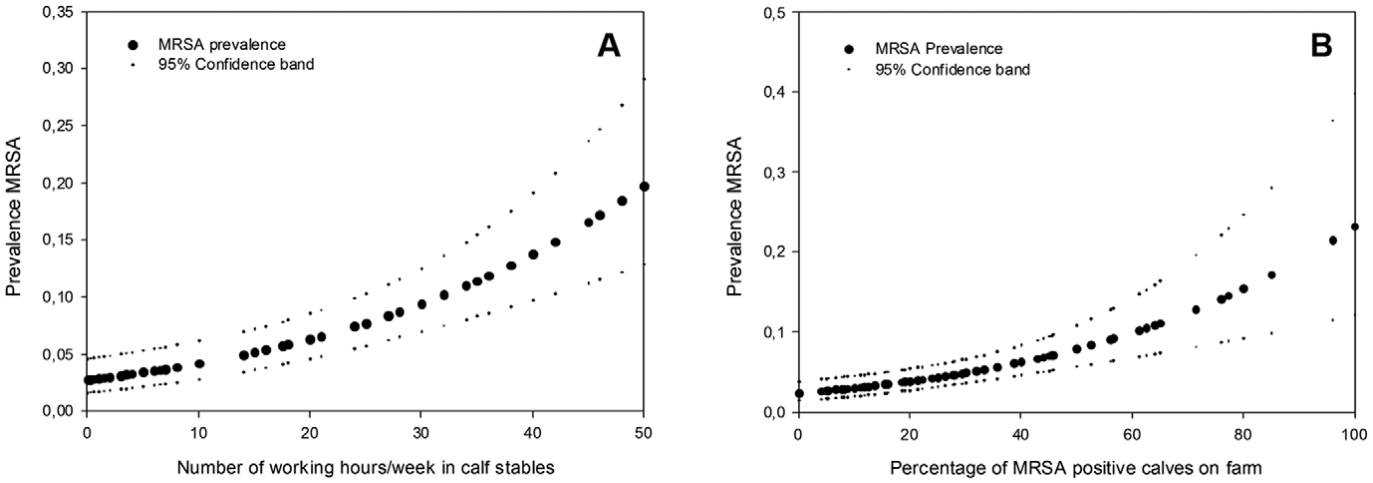
Human MRSA prevalence, working hours in calf stables per week and MRSA positive calves on the farm. Figure 1 A shows the relationship between MRSA carrier prevalence in humans and the number of working hours / week in the calf stable, adjusted for gender, age, smoking habits and percentage of positive calves on the farm. Figure 1 B shows the relationship between MRSA carrier prevalence in humans and the percentage of positive calves on the farm, adjusted for gender, age, smoking habits and number of hours working/week in calf stables. In both figures smoothed plots are given with 95% confidence bands.

Nasal swabs of 390 individuals, working or living on 102 veal calf farms were tested for the presence of MRSA. The response rate was 81%: 97 farmers, 259 family members and 34 employees were included. Reasons for non-participation were no interest, lack of time, or retirement from farming. MRSA prevalence in farmers was 33% and 8% in family members (Table 1). This large difference in MRSA prevalence could be explained by the difference in time spent in the stables, even after adjustment for smoking, age and gender (Table 1). The risk for being MRSA carrier increased with increasing number of working hours spent per week in stables (Odds Ratio (OR) = 1.4, expressed per 10 hours/week). The shape of this relationship was investigated, showing a strong increase in prevalence with increasing time spent with animals (Figure 1A). In addition, those who spent more time on feeding calves (OR = 1.5), veterinary care (OR = 1.6) and stable management (OR = 2.0) were more often MRSA carrier (Table 2).

**Table 1:** Characteristics of 390 farmers, family members, employees and determinants for MRSA carriage in human (multiple logistic regression analysis).

	*No. (Prevalence %)*	
**Total**	390 (16)	
**General charcteristics**		
Farmers	97 (33)	
Family members	259 (8)	
Employees	34 (26)	
**OR (95% Confidence interval) multiple regression model**		
**Gender**		
Female	177 (7)	1
Male	213 (24)	3.0 (1.4–6.8)*
**Age (yr)**		
0–18	130 (6)	
19–65	248 (21)	
66–85	12 (25)	
Per 10 years		1.3 (1.1–1.5)*
**Smoking habits**		
No smoking	334 (17)	1
Smoking	55 (11)	0.5 (0.2–1.4)
**Level of animal contact / # working hours in veal calf stable/week**		
<20 hours a week	250 (7)	
20–40 hours a week	56 (23)	
>40 hours a week	72 (42)	
Per 10 hours/week		1.4 (1.2–1.7)*
**Percentage of positive calves on farm**		
<28% (below mean)	232 (12)	
>28% (above mean)	158 (22)	
Difference between 0–28%		2.1 (1.4–3.0)*

P<0.05.

**Table 2:** Associations between human MRSA carriage and specific tasks on the farm.

Task	MRSA carriage
	OR (95% Confidence interval)^1^ ▴
Feeding calves	1.5 (1.2–1.8)*
Veterinary care	1.6 (1.0–2.5)**
Stable management; sorting calves	2.0 (1.3–3.4)*

*P<0.05.

**P 0.10–0.05.

1 OR expressed per hour/week difference.

▴ adjusted for gender, age, smoking and percentage of positive calves on farm.

MRSA carriage in humans was associated with the prevalence of MRSA in calves. Farmers were more often MRSA carrier when they had more MRSA positive calves. The estimated prevalence in humans was approximately 1% when less than 20% of the calves were carrier. On farms with a higher carrier prevalence in calves MRSA prevalence in humans was above 10%. (Figure 1B). We adjusted for potential confounding variables, like age, gender and smoking habits; age was positively associated (OR = 1.2 per 10 years) and males were significantly more often MRSA colonized compared to females (OR = 3.0). Smoking was negatively associated (OR = 0.5), however not statistically significant. The associations between human MRSA carriage and any of the described determinants (task, duration animal contact, prevalence among animals) did not change when potential confounders were included in the model.

### Calves

**Figure 2:**
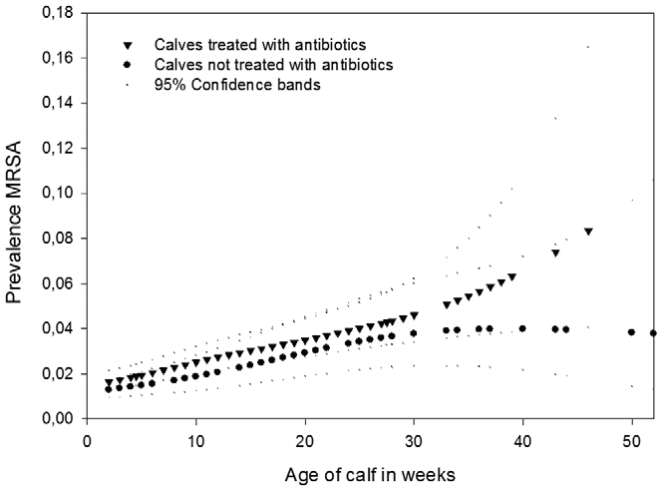
MRSA carriage in veal calves, age and antibiotic treatment. The relationship between MRSA carriage in veal calves and age for calves treated and not treated with antibiotics as group treatment (adjusted for age, number of calves per farm, farm hygiene, calf category (white versus rose veal), number of stables on farm, number of calves per pen, presence of other animals on the farm and rodent control). Smoothed plots are presented with 95% confidence bands.

Nasal swabs were taken from 2151 veal calves of 102 farms (Table 3) and MRSA prevalence was 28%. On 88% of the farms MRSA could be detected in one or more calves. MRSA carriers were more often seen in calves treated with antibiotics (group treatment) (OR = 1.8), compared to calves not treated. Since veal calves were frequently treated with different kinds of antibiotics, even during one treatment, it was not possible to unravel the effect of individual antibiotics or antibiotic classes. Besides this, older calves were more often MRSA positive than calves of younger age (OR = 1.3 (per 10 weeks). The shape of the relationship with age is shown in Figure 2. MRSA carriage with age is higher (p<0.05) in calves treated with antibiotics compared to untreated calves. Calves from large farms (farms with many animals) were significantly more often colonized compared to calves from smaller farms (OR = 2.7 per 500 calves/farm. Moreover, a negative association was found between MRSA carriage and farm hygiene (OR = 0.3). i.e. cleaning of stables before entrance of new calf populations to the farm. Disinfection was applied in less than 20% of the farms and was not associated with MRSA carriage in calves. No associations were found with other possible determinates like country of origin of calves, drinking- or feeding systems and numbers of animals used.

**Table 3:** Characteristics of 2151 veal calves from 102 veal farms and associations between MRSA carriage and some determinants (logistic multiple regression analysis).

	*No. (Prevalence %)*	
**Total**		
**General characteristics**		
Farms	102 (88)	
Veal calves	2151 (28) (range 0–100%)	
**OR (95% Confidence interval) multiple regression model** ▴		
**Antibiotic (group) treatment**		
No	570 (21)	1
Yes	1581 (30)	1.8 (1.1–3.0)*
**Age calf (wk)**		
0–6	301 (12)	
7–12	458 (36)	
>12	1392 (28)	
Per 10 weeks		1.3 (1.1–1.5)*
**Number of calves on the farm**		
<500	985 (22)	
>500	1166 (32)	
Per 100 calves		1.1 (1.0–1.2)*
**Farm hygiene**		
No	375 (35)	1
yes	1776 (26)	0.3 (0.1–0.7)*

*P<0.05.

▴ adjusted for calf category (white versus rose veal), number of stables on farm, number of calves per pen, presence of other animals on the farm and rodent control.

### Genotyping isolates

In total 16 different *spa* types were identified; 9 different *spa* types in human isolates and 12 different types in veal calves (Table 4). In humans, all identified *spa* types belonged to ST398, except four. These four non ST398 strains were identified as t002, t015, t084 and t166), respectively belonging to ST5, ST45, ST15 and CC34.

**Table 4:** Spa typing and MLST typing results (non ST398 sequence types in bold).

*Spa type*	*No. (Prevalence %)*	MLST
**Human isolates n = 62**		
**t002**	**1**	**ST5**
t011	51	ST398
**t015**	**1**	**ST45**
**t084**	**1**	**ST15**
t108	2	ST398
**t166**	**1**	**CC34**
t899	3	ST398
t1457	1	ST398
t2383	1	ST398
**Calve isolates n = 207**		
t011	166	ST398
t034	17	ST398
t108	7	ST398
**t421**	**2**	**ST239**
t899	5	ST398
t1197	1	ST398
**t1236**	**2**	**ST97**
t1451	1	ST398
t1457	1	ST398
t1580	1	ST398
**t1685**	**1**	**ST1159**
t2383	2	ST398
**t3856**	**1**	**CC425**

In calves, the predominant *spa* type found was t011 (80%); also *spa* type t034 was frequently found (8%). Among calves, also four non ST398 strains were found, t421, t1236, t1685, t3856, respectively, ST239, ST97, ST1159 and CC425. All *spa* types found in humans matched with the types found in calves on the same farm except for the non ST398.

## Discussion

This study shows that people in close contact with veal calves have a highly elevated risk of MRSA carriage. Human carriers came more often from farms where veal calves appeared carriers as well. Overall the prevalence of MRSA was 15.9% in persons living and working on a veal calf farm which is a strong elevation compared to the general population. The prevalence of MRSA in the Dutch community is estimated to be below 1%. Moreover, strong associations with duration of animal contact and specific tasks on the farm were found. Furthermore, veal calves carrying MRSA were more often treated with antibiotics. This is the strongest evidence of a direct relationship between antibiotic use in animals and transfer of antimicrobial resistant organisms to humans at present. In addition, this evidence is strengthened by the similarity of *spa* types and antimicrobial susceptibility patterns (data not shown) of the isolates found in both humans and animals.

The presence of LA-MRSA in farmers forms a potential threat for public health, for individuals carrying MRSA and for the health care system. The carriers are at risk for (wound) infections with LA-MRSA. For the health care system, the frequent occurrence of ST398 especially in countries with low and moderate MRSA prevalence put the infection control policy under pressure. In addition, a possible change in virulence (*e.g.* introduction of toxin genes) will change the public health perspective considerably and further challenges the Search and Destroy Strategy. The unexpected and sudden increase of LA-MRSA incidence in hospitals which occurred since 2007, resulted in a shortage of isolation facilities. An additional problem is the treatment of carriers, which is also part of this strategy [26]. Decolonisation of patients who are constantly exposed to MRSA in their work and home settings is not effective: at present the ‘destroy’ element is impossible to execute.

The observation that MRSA carriage in humans is associated with MRSA prevalence among their calves is remarkable. This might indicate that the prevalence in humans in close contact with animals follows the prevalence of MRSA among animals over time. Evidence for an association between the use of antimicrobials in animals and increase in occurrence of resistant bacteria in humans is often not as direct as observed in this study. Parallel occurrence of resistant bacteria in humans and animals is observed in ecological studies. The risk of transfer of resistance from animals to humans is seldom observed directly. For instance, vancomycin-resistant *enterococci* (VRE) acquired their resistance gene (*vanA*) after glycopeptide use as growth promoter in animals and later emerged in hospitals. However, detailed genetic analysis demonstrated that the isolates in animals and humans were not genetically related, and a direct association has never been demonstrated [27]. Similarly, use of fluoroquinolones in poultry co-incided with an increased occurrence of food borne infections in humans with fluoroquinolone resistant *Campylobacter jejuni* [28].

Our results suggest that occurrence of ST398 in humans is transient and varies over time within individuals, because of the correlation with the prevalence in animals. The number of positive calves on the farm is a characteristic that changes over time as shown in this study. Veal calves live between 7–10 months before being slaughtered and new animals are brought in from many different dairy farms, from across the European Union. The prevalence in a new herd of animals is likely to differ from herd to herd. Also the results from fieldworkers who took nasal swabs directly after the farm visits and two days thereafter suggest transient carriage. They were rarely tested MRSA positive during several hundred days of fieldwork but few appeared colonized and when this occurred the duration of colonization was never more than one day (data not shown).

Few studies investigated risk factors for the occurrence of ST398 in humans. High animal to human transmission of ST398 has been reported in pig farming [6], [29]–[32]. In these studies also a large difference in MRSA prevalence is observed between the farmer and family members, which is indicative of a comparable role for intensive animal contact as risk factor for ST398 occurrence [29], [31].

A major finding is that the prevalence of ST398 in veal calves appeared associated with antimicrobial (group) treatments. Also hygiene and some other farm characteristics, such as herd size, were associated with ST398 prevalence. These associations may give further direction to preventive strategies and indicate that is a potential to influence the occurrence of ST398 MRSA in humans and animals.

The large difference in MRSA prevalence between farmers (33%) and family members (8%) may indicate infrequent human to human transmission of ST398. This was also suggested in studies where comparisons were made of ST398 and non-ST398 MRSA regarding the occurrence of secondary cases in hospitals [26], [33]. However, in our study, we did not investigate contact patterns between humans as an explanatory variable. Our observation of a likely low human to human transmission needs further exploration.

MRSA was more frequently observed on veal calf farms as on pig farms [6]. However a larger variety in *spa* types appears present in veal farming, not only in veal calves but also in human isolates. The large number of dairy farms supplying young calves for veal production and their distribution across Europe, and thereby the large variety in origin of veal calves compared to pigs, may play a role in this finding. A limitation of our study is the cross-sectional design in which both cause and effect are measured at the same time. For that reason, results from this study need to be confirmed in a longitudinal study in which occurrence of MRSA carriage over time is associated with the use of antibiotics and other determinants. A longitudinal design may also provide valuable information about dynamics and persistence of MRSA carriage in both animals and humans. Nevertheless, results of this study indicate that prudent use of antibiotics is warranted. This is recognised by all parties responsible for use of antimicrobials in veterinary practice; the Dutch calf producers and the Dutch Government therefore recently agreed on a covenant to reduce antibiotic resistance.

In conclusion, this is the first study showing direct associations between animal and human carriage of ST398. MRSA carriage in calves was positively associated with use of antibiotics while farm hygiene was associated with a lower prevalence of MRSA. For optimal design and implementation of infection control strategies in both animals and humans, detailed combined human-animal studies exploring direct associations are essential. These not only refers to MRSA, but to all (resistant) pathogens in general, where direct associations between animals and humans may be expected.
